# Dermatomyositis Flare-Up Following the SARS-CoV-2 Vaccine: A Case Report and Literature Review

**DOI:** 10.7759/cureus.44324

**Published:** 2023-08-29

**Authors:** Robert Ryad, Alsayed Osman, Ahmad Almusa, Peter Gerges, Bahar Sumbul-Yuksel

**Affiliations:** 1 Internal Medicine, AdventHealth Orlando, Orlando, USA; 2 Internal Medicine, AdventHealth Orlando, Orlando , USA; 3 Rheumatology, AdventHealth Orlando, Orlando , USA

**Keywords:** sars-cov-2, flare-up, vaccine, auto-immune, dermatomyositis, covid 19

## Abstract

Dermatomyositis is a rare auto-immune inflammatory myopathy of unknown etiology. Several environmental factors, including vaccines, have been identified as potential triggers in genetically susceptible individuals. Since the beginning of the coronavirus disease 2019 (COVID-19) pandemic, the development of vaccines (mRNA and vector-based) has been the most effective tool in reducing the incidence, hospitalization rates, and mortality of COVID-19. However, among individuals with immune dysregulation and auto-immune conditions, unique challenges may arise with immune stimulation. We present a case of a dermatomyositis flare-up following severe acute respiratory syndrome coronavirus 2 (SARS-CoV-2) vaccination. A 40-year-old Hispanic female presented to the emergency department with shortness of breath, muscle pain and weakness, and skin rash for two days. She had been recently diagnosed with dermatomyositis six months prior based on clinical presentation, laboratory investigations, and characteristic muscle biopsy findings. She had been on treatment with mycophenolate mofetil, prednisone, and hydroxychloroquine since. She reported receiving the second dose of the BNT162b2 COVID-19 vaccine one day prior to the onset of symptoms. Physical examination revealed erythematous plaques over her cheeks, upper chest, and arms, in addition to Gottron papules on her hands. She had reduced proximal muscle strength and scattered dry crackles bilaterally on lung auscultation. Her laboratory investigations were remarkable for elevated erythrocyte sedimentation rate, C-reactive peptide, creatinine kinase, and troponin T. The SARS- CoV-2 PCR test was negative. CT scan of the chest showed evidence of pneumonitis. A diagnosis of the dermatomyositis flare-up potentially secondary to the SARS-CoV-2 BNT162b2 vaccine was established. The patient was admitted and treated with pulse steroids and intravenous immunoglobulin. She responded well to therapy and was discharged home four days later. There have been several reports of a new onset of dermatomyositis following the SARS-CoV-2 vaccine which highlights the need for further large-scale studies to estimate the prevalence of such adverse effects. The benefits of the SARS-CoV-2 vaccine outweigh the risks even among patients with auto-immune and rheumatologic conditions; however, it is important for clinicians to recognize the possibility of occurrence of such events in order to manage patients appropriately.

## Introduction

The coronavirus disease 2019 (COVID-19) pandemic has been responsible for the deaths of more than 1,000,000 people in the US alone and continues to mount significant challenges to our healthcare system [1]. The development of the severe acute respiratory syndrome coronavirus 2 (SARS-CoV-2) vaccine has been the most effective tool in reducing the incidence, hospitalization rate, and mortality of the disease in the general population [2]. However, as with all medical interventions, it is important to recognize complications and adverse reactions to continuously guide our clinical approach and decision-making. More so in patients with immune dysregulation and auto-immune conditions who usually require a more tailored approach depending on their disease status, co-morbid conditions, and risk for severe disease and opportunistic infections. Herein, we report a case of a dermatomyositis flare-up following the SARS-CoV-2 vaccine and perform a brief literature review on the current state of knowledge regarding COVID-19 infection and vaccination in relationship to auto-immune connective tissue diseases.

## Case presentation

**Figure 1:**
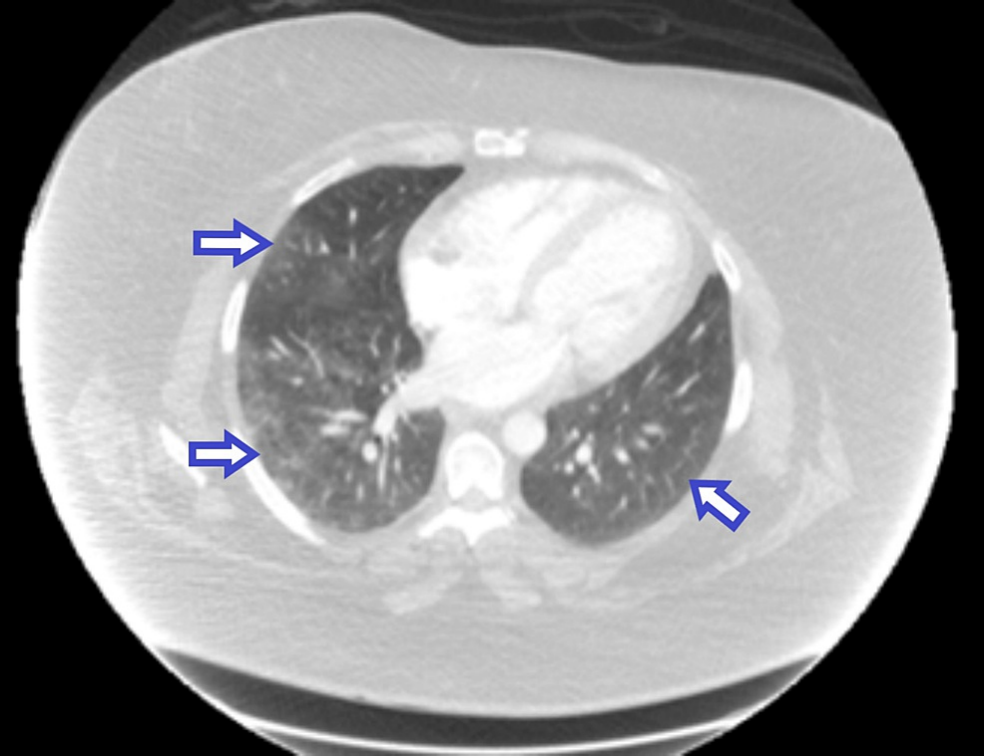
Chest CT scan with IV contrast showing bilateral ground glass opacities concerning pneumonitis as highlighted by the arrows

A 40-year-old Hispanic female presented to the emergency department complaining of shortness of breath, muscle pain and weakness, and skin rash for two days. She had recently been admitted to the hospital six months prior for similar complaints, in addition to hemoptysis. She underwent extensive workup at the time including a muscle biopsy which showed evidence of dermatomyositis and a bronchoscopy which revealed diffuse alveolar hemorrhage. Following with the rheumatology department since then, she had been receiving mycophenolate mofetil, hydroxychloroquine, and prednisone and had been mostly asymptomatic until two days ago. She reported that she received the second dose of the Pfizer BioNTech (BNT162b2) COVID-19 vaccine one day prior to the onset of symptoms. Her other medical conditions included essential hypertension and controlled hypothyroidism for which she was receiving metoprolol and levothyroxine, respectively. She affirmed strict compliance with all her medications. Family history was negative for auto-immune or connective tissue disease. She had a remote smoking history but denied alcohol or illicit drug use. On physical examination, her heart rate was 90, blood pressure 172/93, respiratory rate 20, oxygen saturation 98% on room air, and temperature 98.0. Lung auscultation revealed scattered dry crackles bilaterally. She had bilateral proximal muscle tenderness and reduced strength. Her skin examination showed erythematous plaques over her cheeks, upper arms, and chest, as well as Gottron papules in her hands. Her laboratory investigations, as shown in Table 1, were significant for elevated erythrocyte sedimentation rate (ESR), C-reactive peptide (CRP), creatinine kinase (CK), and troponin levels. SARS-CoV-2 PCR was negative. The respiratory PCR panel was negative for any other viral infections. Urinalysis was unremarkable. CT scan of the chest with IV contrast showed bilateral patchy ground glass opacities, concerning pneumonitis as seen in Figure 1. Based on the above findings, the patient was diagnosed with a dermatomyositis flare-up potentially secondary to the COVID-19 vaccine. She was admitted and treated with high-dose IV Solu-Medrol and intravenous immunoglobulin (IVIG) for a total of four days. She showed continuous improvement while in the hospital and was eventually discharged on a tapering steroid regimen in addition to her previous maintenance medications. She remained in stable condition upon follow-up with her rheumatologist in the outpatient clinic after discharge.

**Table 1:** Laboratory investigations upon current presentation and initial presentation six months prior

Laboratory Investigations	Reference Range	Current presentation	Initial presentation six months prior
Total leukocyte count, 10^9 ^/L	4.5-10.5	6.13	5.92
Hemoglobin concentration, g/dL	11.4-14.7	12.2	11.2
Platelet count, 10^9 ^/L	140 – 360	355	323
Serum creatinine, mg/dL	0.60–1.20	10	9
Blood urea nitrogen, mg/dL	5-25	0.61	0.70
Alanine aminotransferase (ALT), units/L	4-51	51	150
Aspartate aminotransferase (AST), units/L	5-46	114	307
Alkaline phosphatase (ALP), units/L	35-104	112	127
Creatinine kinase (CK), units/L	24-200	2,950	3,998
Troponin T, units/L	<0.03	0.36	1.06
Aldolase, units/L	1.5-8.1	-	36.2
Erythrocyte sedimentation Rate (ESR), mm/hr	0-20	58	69
C-reactive protein (CRP), mg/L	<5	14.0	33.9
Antinuclear antibody (ANA)		-	Negative
Rheumatoid factor (RF)		-	Negative
Anti-Jo-1 antibody	<1.0	-	<0.2; Negative
Anti SCL-70 antibody	<1.0	-	<0.2; Negative

## Discussion

Dermatomyositis is a rare autoimmune idiopathic inflammatory myopathy that commonly affects women more than men. It usually presents with symmetrical muscle weakness, skin involvement, and elevated muscle enzymes at any age [3]. The exact etiology remains unknown; however, several environmental factors are believed to be a triggering point in a genetically susceptible population, and viruses and vaccines were reported to be among those factors [4,5]. Vaccine-induced autoimmunity remains a subject of ongoing research; however, the currently proposed mechanisms are vaccine adjuvants and molecular mimicry between the host cell and antigen [6,7]. 

The development of the SARS-CoV-2 vaccine has been the most effective tool in reducing the incidence of COVID-19, hospitalization rate, and mortality of the disease in the general population. mRNA COVID-19 vaccines induce a T-cell-mediated immune response to a protein that has been translated from the mRNA which expresses a significant level of immunity [8]. Several case reports have linked COVID-19 vaccines to potential side effects. The most prevalent adverse effects were injection site pain, fatigue, headache, myalgia, chills, arthralgia, and fever. Life-threatening side effects like Guillain-Barre syndrome, myocarditis, and pericarditis were sporadically reported [9,10]. Inflammatory myositis including dermatomyositis has been reported following COVID-19 vaccination; however, the causal relationship remains an area of research given the rarity of these cases [11-16]. BNT162b2 mRNA COVID-19 vaccination safety in patients with rheumatic and musculoskeletal diseases is comparable to the general population [17]. To the best of our knowledge, there have been no reports of vaccine-triggered dermatomyositis flare-ups.

## Conclusions

The benefits of the SARS-CoV-2 vaccine outweigh the risks even among patients with auto-immune and rheumatologic conditions; nonetheless, it is important for clinicians to recognize the possibility of such rare events in order to manage patients appropriately. Further studies are needed to estimate the incidence of such events and develop management strategies to prevent their occurrence.
